# Repetitive DNA in the Architecture, Repatterning, and Diversification of the Genome of *Aegilops speltoides* Tausch (Poaceae, Triticeae)

**DOI:** 10.3389/fpls.2018.01779

**Published:** 2018-12-04

**Authors:** Yulia Pollak, Einat Zelinger, Olga Raskina

**Affiliations:** ^1^The CSI Center for Scientific Imaging, The Robert H. Smith Faculty of Agriculture, Food and Environment, Hebrew University of Jerusalem, Rehovot, Israel; ^2^The Electron Microscopy Unit, Faculty of Natural Science, University of Haifa, Haifa, Israel; ^3^Institute of Evolution, University of Haifa, Haifa, Israel

**Keywords:** *Aegilops speltoides*, interphase nuclei, nonhomologous recombination, repetitive DNA, somatic recombination, tandem repeats

## Abstract

The genome’s adaptability to environmental changes, especially during rapid climatic fluctuations, underlies the existence and evolution of species. In the wild, genetic and epigenetic genomic changes are accompanied by significant alterations in the complex nuclear repetitive DNA fraction. Current intraspecific polymorphism of repetitive DNA is closely related to ongoing chromosomal rearrangements, which typically result from erroneous DNA repair and recombination. In this study, we addressed tandem repeat patterns and interaction/reshuffling both in pollen mother cell (PMC) development and somatogenesis in the wild diploid cereal *Aegilops speltoides*, with a focus on genome repatterning and stabilization. Individual contrasting genotypes were investigated using the fluorescent *in situ* hybridization (FISH) approach by applying correlative fluorescence and electron microscopy. Species-specific Spelt1 and tribe-specific Spelt52 tandem repeats were used as the markers for monitoring somatic and meiotic chromosomal interactions and dynamics in somatic interphase nuclei. We found that, the number of tandem repeat clusters in nuclei is usually lower than the number on chromosomes due to the associations of clusters of the same type in common blocks. In addition, tightly associated Spelt1–Spelt52 clusters were revealed in different genotypes. The frequencies of nonhomologous/ectopic associations between tandem repeat clusters were revealed in a genotype-/population-specific manner. An increase in the number of tandem repeat clusters in the genome causes an increase in the frequencies of their associations. The distal/terminal regions of homologous chromosomes are separated in nuclear space, and nonhomologous chromosomes are often involved in somatic recombination as seen by frequently formed interchromosomal chromatin bridges. In both microgametogenesis and somatogenesis, inter- and intrachromosomal associations are likely to lead to DNA breaks during chromosome disjunction in the anaphase stage. Uncondensed/improperly packed DNA fibers, mainly in heterochromatic regions, were revealed in both the meiotic and somatic prophases that might be a result of broken associations. Altogether, the data obtained showed that intraorganismal dynamics of repetitive DNA under the conditions of natural out-crossing and artificial intraspecific hybridization mirrors the structural plasticity of the *Ae. speltoides* genome, which is interlinked with genetic diversity through the species distribution area in contrasting ecogeographical environments in and around the Fertile Crescent.

## Introduction

In the wild, the genome’s ability to adapt to changing environments, especially in a period of significant climatic changes, underlies the existence of the species and its evolution (Grant, 1981; Tchernov, 1988). In plant populations, genetic changes and epigenetic modifications are accompanied by significant diversification in the abundances and patterns of repetitive DNA, which is the prevailing genomic fraction in cereals (Bennetzen, 1996; Feschotte et al., 2002). Transposable elements (TEs) and tandem repeats compose the largest portion of the genome of wild diploid grass species *Aegilops speltoides* (sect. Sitopsis; 2n = 2x = 14) (Middleton et al., 2013). *Ae. speltoides* is a dimorphic species; the differences in spike morphology are controlled through a block of closely linked genes encoding dominant ssp. *ligustica* and recessive ssp. *aucheri* morphotypes, which coexist in mixed panmictic populations with different ratios (Zohary and Imber, 1963; Kimber and Feldman, 1987). Plants with intermediate *ligustica/aucheri* phenotypes have also been observed in the wild, suggesting genetic changes in the linked group of genes (Belyayev and Raskina, 2013). *Ae. speltoides* is only out-crossing species among the five diploid species of the sect. Sitopsis; however, under a changing environment, specifically, in drought conditions, *Ae. speltoides* transits to self-pollination, which is an extremely rare phenomenon in the plant kingdom (Zohary and Imber, 1963). The plasticity of the genome underlies a wide range of *Ae. speltoides* distribution and adaptability to contrasting ecogeographical environments in and around the Fertile Crescent (Zohary and Imber, 1963; Kimber and Feldman, 1987). Thus, at the northern periphery of the species distribution area, Turkish winter-type populations have a long vegetative cycle and exhibit a specific morphology, which significantly distinguishes them from the peripheral/marginal southern Israeli populations (Belyayev and Raskina, 2013). In parallel, the *Ae. speltoides* genome is characterized by high intraspecific polymorphism in abundance and patterns of different types of repetitive DNA, specifically, TEs (Middleton et al., 2013; Yaakov et al., 2013) and tandem repeats (Badaeva et al., 1996; Raskina et al., 2011; Molnár et al., 2014; Raskina, 2017), which underlie permanent intraorganismal and intraspecific genome reshuffling (Belyayev et al., 2010; Shams and Raskina, 2018). The current intraspecific polymorphism and intraorganismal dynamics of the highly repetitive DNA fraction in the genome of *Ae*. *speltoides* is largely caused by ongoing chromosomal rearrangements, which are typical results of erroneous DNA repair and recombination (Andersen and Sekelsky, 2010; Knoll et al., 2014; Zeman and Cimprich, 2015).

In the present research, we addressed repetitive DNA dynamics in the *Ae. speltoides* genome, both during pollen mother cell (PMC) development and in somatogenesis, with a focus on genome repatterning and stabilization. We traced tandem repeats’ reshuffling/interactions during the cell cycle using fluorescent *in situ* hybridization (FISH), applying correlative fluorescence and electron microscopy. Species-specific Spelt1 (Salina et al., 1998) and tribe-specific Spelt52 (Anamthawat-Jónsson and Heslop-Harrison, 1993) tandem repeats were used as the markers for monitoring somatic and meiotic chromosomal interactions and dynamics in somatic interphase nuclei. We found that Spelt1 and Spelt52 demonstrated sequence-specific and genotype-/population-specific abundances and dynamics in interphase nuclei. The number of tandem repeat clusters in nuclei is usually lower than the number on chromosomes due to the associations of clusters of the same type in common blocks. In addition, tightly associated Spelt1–Spelt52 clusters were revealed in different genotypes. An increase in the number of tandem repeat clusters in the genome causes an increase in the frequencies of their associations in common blocks in interphase nuclei. Frequent cell-specific interchromosomal somatic associations and nonhomologous recombination in microsporogenesis were revealed. It is speculated that significant number of nonhomologous chromosomal associations detected in microgametogenesis might be the consequences of cell-specific ectopic recombination events that occurred in premeiotic cell lineages.

## Materials and Methods

### Plant Material

Original plants of *Ae. speltoides* from contrasting allopatric populations Cankiri (Turkey; PI 573448, USDA), Ankara (Turkey; PI 573452, USDA), Katzir (Israel; 2.93, Institute of Evolution University of Haifa), Ramat haNadiv (Israel; 2.46, Institute of Evolution University of Haifa), and artificial F_1_–F_2_ intraspecific hybrids (Raskina, 2017), were analyzed.

### Preparation of Chromosomal Spreads

Anthers containing PMC cells at the pachytene-diakinesis stages and seedling shoot apical meristems of individual plants were used for meiotic and mitotic chromosome spreads, respectively. Seeds were germinated on moist filter paper at 24°C in the dark. Seedlings 5–7 mm length were transferred to ice water for 24–26 h to accumulate metaphases and then fixed in 3:1 (v/v) 100% ethanol:acetic acid. The procedure of chromosome spread preparation has previously been described (Raskina et al., 2004). Specifically, the fixed seedlings and anthers were washed (3 × 5 min) in water and then incubated in an enzyme buffer (10 mM citrate buffer at pH 4.6) and partially digested (meristem–for 50 min, anthers–for 30 min) in 6% pectinase plus 0.5% cellulase (NBC Biomedicals, United Kingdom) plus 5% cellulase “Onozuka” R-10 (Yakult Honsha Co., Ltd.) followed by washes in enzyme buffer (3 × 5 min) and distilled water (3 × 5 min). The material, in a drops of water, was transferred onto a grease-free microscope slide, and the cells were spread with a metal stainless needle in the drop of 60% acetic acid at 45–47°C on the hot plate, then fixed in 3:1 (v/v) 100% ethanol : acetic acid, and then immersed in absolute ethanol for 3–5 s. Dry chromosome spreads were used for *in situ* hybridization.

### *In situ* Hybridization Procedures

For the FISH experiments, cytological slides of individual anthers and seedling shoot apical meristems containing well-spread chromosomal plates were used. The FISH procedures were conducted as previously described (Shams and Raskina, 2018).

Cells were treated with 1 μg/mL of DNase-free RNase A in 2 × SSC (0.3 M NaCl plus 30 mM trisodium citrate) for 60 min at 37°C followed by 3 × 3 min washes with 2 × SSC at 37°C. The preparations were then dehydrated in an ethanol series (70, 90, and 100%, 3 min each) at room temperature, washed 2 × 2 min with 2 × SSC, and then allowed to air-dry. The hybridization mixture (20 μL per slide under the glass coverslip 22 mm × 22 mm) contained 10% dextran sulfate, 2 × SSC, and 50 ng each of DNA probe. DNA probes and chromosome spreads were simultaneously denatured at 95°C for 3 min and hybridized using ThermoBrite StatSpin System (Abbott, United States). Hybridization was carried out at 63°C for 2 h. After removal of the coverslips in 2 × SSC at 63°C, the slides were washed for 2 × 5 min in 2 × SSC at 63°C, additionally once in 0.1 × SSC for 5 min at 63°C to increase stringency, then cooled to 37°C and washed for 2 × 5 min in 0.1 × SSC; cooled to room temperature, washed in distilled water for 1 min, allowed to air-dry for 20 min, and mounted in VECTASHIELD antifade mounting medium (Vector Laboratories).

Tandem repeats Spelt1 (Salina et al., 1998), Spelt52 (Anamthawat-Jónsson and Heslop-Harrison, 1993), pSc119.2 (Bedbrook et al., 1980), pTa71 (for the localization of 45S rDNA) (Taketa et al., 2000), and As5SDNAE (for the localization of 5S rDNA) (Baum and Bailey, 2001) were used as the DNA probes for FISH. The DNA probes were directly labeled with Cy-3, Fluorescein-12-dUTP, and ATTO-425 (Jena Bioscience, Germany). AT-specific 4′,6-diamidino-2-phenylindole (DAPI) fluorochrome was used for differential staining.

### Epi-Fluorescence Imaging

The slides were examined on a Leica DMR fluorescent microscope equipped with a DFC300 FX CCD color camera using following filter sets: A for DAPI, I3 for Fluorescein-12-dUTP, N2.1 for Cy3, FI/RH for Fluorescein-12-dUTP/Cy3, and B/G/R for blue/green/red fluorescence.

### Confocal Image Acquisition and Analysis

Confocal imaging was done using a LEICA SP8 (CTR6000) microscope with a Leica HC PL APO CS2 × 63 N.A 1.4 oil objective. Lasers used: EX 405 EM 430–470 for DAPI, EX 488 EM 500–540 for Fluorescein-12-dUTP, and EX 552 EM 560–590 for Cy3. All images were collected with the HyD (hybrid detector). Image z-stacks of 15–25 × 0.2 μm slices per specimen were acquired and reconstructed by the 3D built-in module of LEICA SP8 in the LAS AF software (Supplementary Figures S1–S3). 3D Movies were created from the z-stacks by the LEICA SP8 3D module (Supplementary Movies S1–S4). For statistical analysis and 3D reconstruction the ImageJ software^1^ was used (Supplementary Figure S3B).

For 3D reconstruction modeling, the images were processed using the Imaris surface reconstruction tool with module “Imaris cell” (Bitplane Scientific Software, Zurich, Switzerland) (Supplementary Figure S4).

### Scanning Electron Microscopy

The scanning electron microscopy (SEM) images were recorded using Field emission scanning electron microscopes (i) Sigma-HD, ZEISS, and (ii) JEOL, JSM-7800F, accelerating voltage of 1.5–2.0 kV. Slides were carbon coated using Quorum Q150T.

## Results

### Patterns of Tandem Repeats on Chromosomes and in Somatic Interphase Nuclei in Plants From the Cankiri Population

**FIGURE 1:**
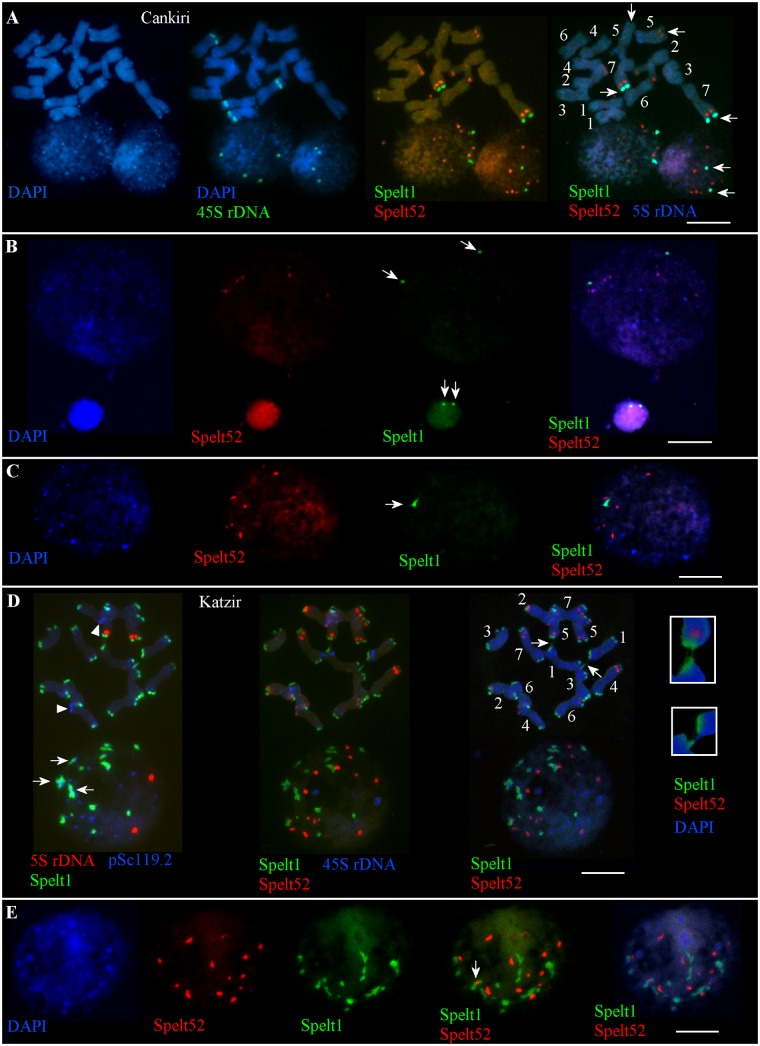
Patterns of tandem repeats in the *Ae. speltoides* contrasting genotypes. Fluorescent *in situ* hybridization (FISH) on somatic chromosomes and interphase nuclei from the seedling shoot apical meristems of the plants from Cankiri **(A–C)** and Katzir **(D,E)** populations. DNA probes for FISH: Spelt1 (in green), Spelt52 (in red), pSc119.2 (in blue), 5S rDNA (in blue, red), and 45S rDNA (in green). **(A)** Somatic chromosomes **(top)** and interphase nuclei **(bottom)** of the plant from the Cankiri population. Two large terminal Spelt1 clusters on the long arms of homologous chromosome 7 and two small terminal clusters on the long arms of homologous chromosome 5 were revealed (arrows). Nine Spelt52 clusters were detected in the diploid genome. In the interphase nuclei, Spelt52 and two large Spelt1 (arrows) clusters are separated from each other. Four separated 45S rDNA clusters are revealed in the nuclei. **(B)** Two large Spelt1 clusters in the interphase nuclei of different sizes are indicated with arrows. **(C)** Single Spelt1 cluster (arrow) and separated Spelt52 clusters are observed in the interphase nucleus of Cankiri plant. **(D)** Tandem repeats Spelt1 and pSc119.2 form complex clusters in chromosome termini **(top left)**. There are 26 Spelt1 clusters in the diploid genome; 12 chromosomes carry the Spelt1 cluster in both arms and two chromosomes carry Spelt1 in one arm, with terminal pSc119.2 clusters shown in the other arm with arrowheads. In the interphase nucleus **(bottom)**, 14 Spelt1 clusters are revealed due to the association of individual clusters. In addition, Spelt1 and pSc119.2 tandem repeats compose common clusters (arrows). Two 5S rDNA clusters are separated in the nuclear space. Re-probing with Spelt52 and 45S rDNA **(middle)** revealed 14 Spelt52 clusters on the chromosomes and in the nucleus. Ectopic chromatin fibers between nonhomologous chromosomes (arrows; enlargement in the small boxes) are indicated with arrows **(right)**. **(E)** Interphase nucleus of the Katzir genotype. Spelt52 clusters are separated in the nuclear space, while the number of Spelt1 clusters is almost half that on the chromosomes. Tightly associated Spelt1 and Spelt52 clusters are indicated with arrow. Scale bar = 10 μm.

There are nine Spelt52 clusters of different sizes and fluorescence intensity, two large Spelt1 clusters on the long arms of the both homologs of chromosome 7, and two small Spelt1 clusters on the long arms of both homologs of chromosomes 5 in the diploid genome(s) of plant(s) from the Cankiri population (Figure 1A). The number of Spelt52 clusters in the interphase nuclei varied between six and nine. Among 300 interphase nuclei of different sizes and varying degrees of chromatin compactness analyzed on two slides from the seedling shoot apical meristems, two large Spelt1 clusters were observed in most cases (Figures 1A,B) and only in eight nuclei (2.7%) was a single Spelt1 cluster revealed (Figure 1C). Thus, these data point to separation of the homologous chromosomes in nuclear space; associations of the distal/terminal chromosomal regions and the formation of complex tandem repeat clusters rarely occurred.

### Patterns of Tandem Repeats on Chromosomes and in Somatic Interphase Nuclei in Plants From the Katzir Population

In contrast to the genotypes from the Cankiri population, plants from the Katzir population are enriched with Spelt1 tandem repeats (Figures 1D,E). There are 14 Spelt52 and 26 large terminal Spelt1 clusters in the diploid genome; only two chromosome arms do not carry Spelt1 clusters (Figure 1D, top); instead, clusters of the pSc119.2 tandem repeat are detected in these positions. The genome of *Ae. speltoides* is enriched with the pSc119.2 tandem repeat, which forms numerous clusters in the distal/terminal and intercalary chromosomal regions (Badaeva et al., 1996; Molnár et al., 2014). In the interphase nuclei, the Spelt1 and pSc119.2 tandem repeats form common clusters (Figure 1D). The number of Spelt1 clusters is reduced almost by half due to associations, while the number of Spelt52 clusters equals the number of clusters on the chromosomes, and 5S rDNA clusters are separated in the nuclear space. Among the 130 nuclei from the seedling shoot apical meristems analyzed on this cytological slide, the number of Spelt1 clusters was reduced in most cases, and one to three tightly associated Spelt1–Spelt52 clusters were revealed in 123 (94.6%, *n* = 130) nuclei (Figures 1D,E); and in other genotype, colocalization of the Spelt1–Spelt52 clusters was revealed in most cases (78.5%, *n* = 250).

### Patterns of Tandem Repeats on Chromosomes and in Somatic Interphase Nuclei in Plants From the Ramat haNadiv Population

**FIGURE 2:**
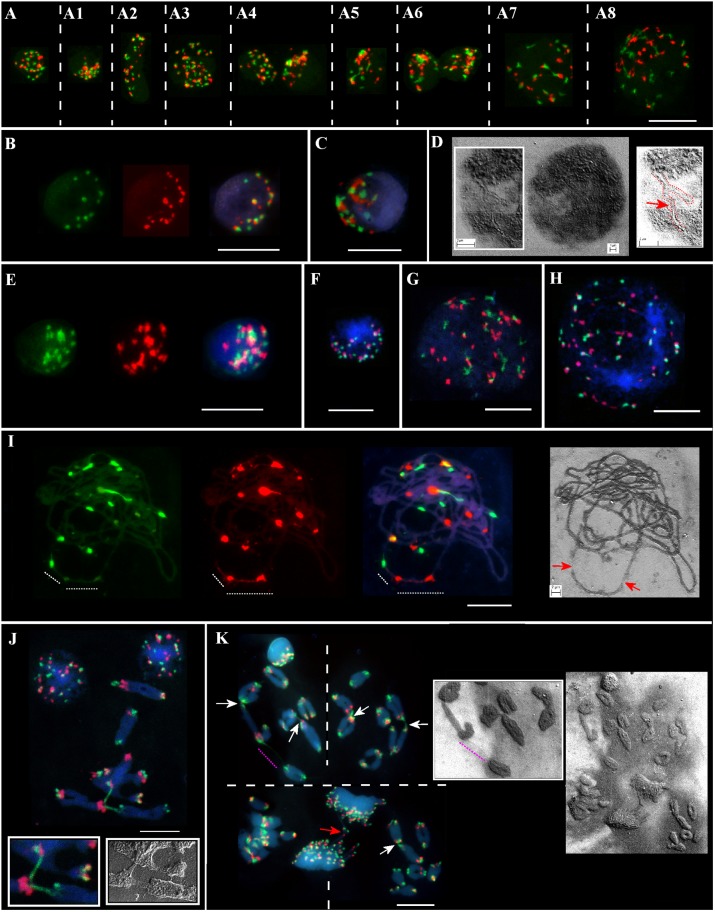
Patterning of Spelt1 and Spelt52 tandem repeats on meiotic chromosomes and in interphase nuclei from the anther’ somatic tissues in individual genotypes of *Ae. speltoides* from the Ramat haNadiv population. DNA probes for FISH: Spelt1 (in green) and Spelt52 (in red); counterstaining with DAPI (in blue). **(A)** Interphase nuclei at different interphase stages belonging to different anther somatic tissues from the same cytological slide. **(A–A8)** (Supplementary Figures S1,S2 and Supplementary Movies S1,S2) In nuclei of different sizes and shapes, various numbers of Spelt1 and Spelt52 clusters are observed. The nuclei differ in number and sizes of associated clusters of the same types and Spelt1–Spelt52 colocalized clusters. Condensed and undercondensed single and associated Spelt1 and Spelt52 tandem repeat clusters are revealed in different nuclei. A polyploid nucleus is shown in **A3**. **(A6)** Amitotically dividing nuclei. **(A7,A8)** Nuclei at the late interphase–early prophase stages. Spelt1 and Spelt52 clusters are partially decondensed and interconnected with extended fibers; due to associations, the cluster number is less than on meiotic chromosomes **(J,K)**. **(B–D)** Nuclei from the same cytological slide. **(B)** Number of condensed Spelt1 and Spelt52 clusters of different sizes and fluorescent intensities are 11 and 17, respectively. Most Spelt1 and Spelt52 clusters associate in common clusters. **(C)** Large Spelt1 and Spelt52 clusters aggregate in complex blocks. **(D)** In the image of the interphase nucleus obtained using scanning electron microscopy, two interconnected Y-shaped chromatin structures are revealed. Chromatids of two different chromosomes form a double-strand chain (red dashed line and arrow in the scheme on the right). **(E–H)** Nuclei of the other genotype from the same cytological slide. **(E)** (Supplementary Figures S3,S4 and Supplementary Movies S3,S4) Large Spelt1 and Spelt52 partially decondensed clusters aggregate in complex blocks. **(F)** Highly condensed clusters of Spelt1 and Spelt52 form common blocks. **(G,H)** Large nuclei at the late interphase–early prophase stages; condensed chromatid fibers are revealed. Spelt1 and Spelt52 clusters interconnected with extended fibers. **(I)** Meiotic prophase I, pachytene stage: homologous synapsis is completed. Associated Spelt1 and Spelt52 clusters form common blocks, and extended fibers between clusters are observed; improper condensation and damaged chromatin fibers are indicated with dashed lines and arrows on the image obtained via scanning electron microscopy **(right)**. **(J,K)** Meiotic chromosomes and nuclei of different genotypes. Numbers of Spelt52 vary from 11 to 12 clusters and Spelt1 from 13 to 14 clusters per seven bivalents. **(J)** Three bivalents are involved in nonhomologous recombination. Extended Spelt1 fibers connect two homologs of one bivalent with single chromosomes of two other bivalents. In two **small boxes**, enlargement of this region obtained with confocal fluorescent **(left)** and scanning electron microscopy **(right)**, respectively, is shown. **(K)** Four meiotic chromosomal plates at the diakinesis–metaphase I stages and dividing polyploid cell of tapetum at the anaphase stage with chromosomal bridges (red arrow). Images were taken consistently using fluorescent and scanning electron microscopes. Nonhomologous chromosome associations are indicated with arrows; the extended thin Spelt1 fiber between two bivalents is indicated with a pink dashed line. Scale bar = 10 μm.

Meiotic chromosomes and interphase nuclei from the somatic anther tissues were analyzed on the same slide(s) for different genotypes from the Ramat haNadiv population (Figure 2). At the early stages of anther’ development, specifically, at the pachytene–diakinesis–metaphase I stages, the following cell layers, namely epidermis, endothecium, middle layer, tapetum, and connective tissues are distinguished (Browne et al., 2018). The nuclei of different sizes, shapes, and chromatin compactness at different stages of the interphase from the anther’s somatic tissues vary significantly in the numbers of Spelt1 and Spelt52 clusters (Figures 2A–A8, Supplementary Figures S1,S2, and Supplementary Movies S1,S2). Colocalization of the tandem repeat clusters of the same type is observed in a cell-specific manner; in addition, Spelt1 and Spelt52 tandem repeats form tightly associated/common clusters (100%; *n* = 400; three slides/genotypes), which are condensed or partially decondensed at different interphase stages and in different anther somatic tissues. Numerous polyploid (Figure 2A3) and amitotically dividing tapetal nuclei (Figure 2A6) are revealed on the same cytological slide(s). Typically, partial decondensation of tandem repeat clusters and the occurrence of large common clusters are observed in small compact nuclei (Figures 2A4–A6,C,E, Supplementary Figures S3,S4, and Supplementary Movies S3,S4). Somatic recombination in the interphase nucleus was documented using a SEM (Figure 2D). Specifically, two chromatin forks/two different chromosomes are recombined in their terminal regions, which form continuous chromatin fiber. At the end of the interphase/in the early somatic prophase stage, as condensed chromatin fibers become visible, numerous associations between clusters of the same type and between Spelt1 and Spelt52 clusters are still observed (Figures 2A7,A8, G,H).

### Nonhomologous Chromosome Associations in Microsporogenesis in Different Genotypes From the Ramat haNadiv Population

Along with somatic interphase nuclei from the anther tissues, meiotic cells were analyzed on the same slide(s). A wide spectrum of cell-specific structural alterations and chromosomal rearrangements at the meiosis I stages were revealed in different genotypes (Figures 2I–K). Thus, alterations in Spelt1 and Spelt52 clusters’ condensation and the appearance of tightly associated clusters were observed in late meiotic prophase I (Figure 2I); chromatin fibers appear damaged and uncondensed. At the stages of diakinesis–metaphase I, cell-specific chromosomal rearrangements and numerous nonhomologous associations were revealed in all plants (Figures 2J,K). Interchromosomal fibers were documented using both fluorescent and scanning electron microscopy.

### Tandem Repeat Cluster Associations in Somatic Interphase Nuclei in Parental Genotype From the Katzir Population and Intraspecific Hybrids of *Aegilops speltoides*

**FIGURE 3:**
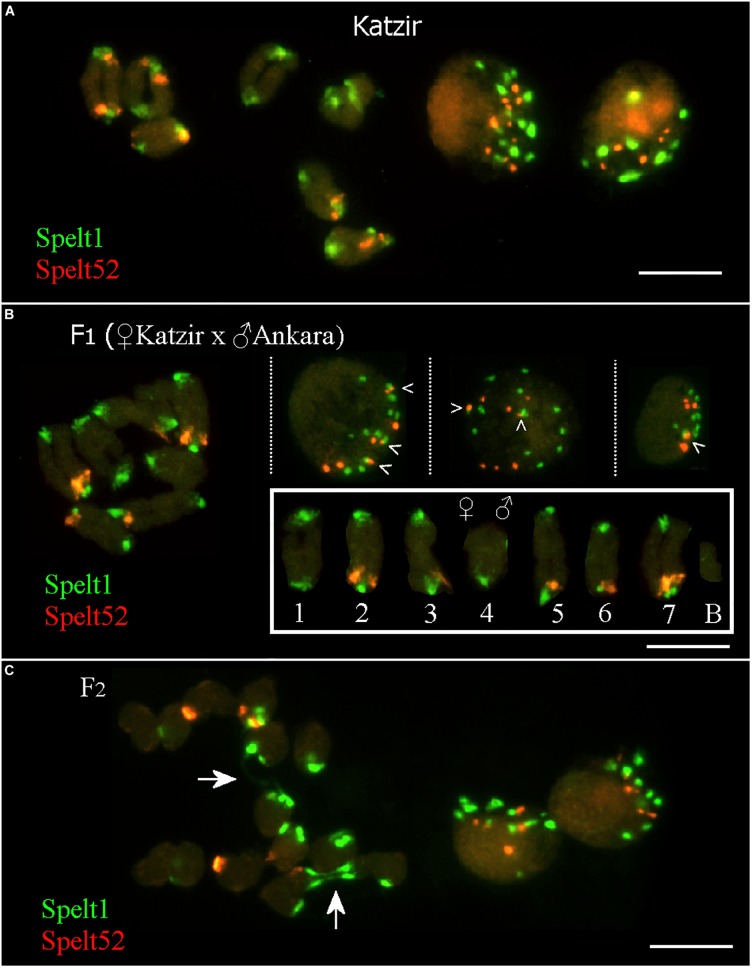
Fluorescent *in situ* hybridization (FISH) with Spelt1 and Spelt52 tandem repeats on meiotic chromosomes and interphase nuclei from the anther’ somatic tissues of individual genotypes of *Ae. speltoides.* **(A)** In the genotype from the population Katzir, there are 22 Spelt1 clusters on 14 individual chromosomes and 14 clusters per 7 bivalents. There are 12 Spelt52 clusters on individual chromosomes, and seven clusters per seven bivalents. Two interphase nuclei differ in the abundance of tandem repeats clusters: there are 13 Spelt1 and 10 Spelt52 clusters in one nucleus **(left)**, whereas 8 clusters of Spelt1 and 5 clusters of Spelt52 are revealed in the other nucleus **(right).** **(B)** In the hybrid F1 genotype (2n = 2x = 14 + B) obtained in the crosses between plants from the Katzir and Ankara populations, there are 15 Spelt1 clusters (12 from the maternal genome and 3 from the paternal genome) and 7 Spelt52 clusters (two from Katzir genotype and five from Ankara genotype) on 14 individual chromosomes (**small box;** the orientation of bivalents corresponds to the position of the maternal chromosomes on the left and paternal chromosomes on the right). In terms of the number of clusters per seven bivalents, there are 13 Spelt1 (both homologous chromosomes 4 do not carry Spelt1 in the short arms) and 5 Spelt52 (in the long arms of one or two conjugated homologous chromosomes 2, 3, 5, 6, and 7) clusters. In three different interphase nuclei from the same cytological slide **(top)**, there are 12 Spelt1 and 8 Spelt52 clusters in one nucleus **(left)**; the same numbers are revealed in the second nucleus **(middle)**, and 8 Spelt1 and 5 Spelt52 clusters were detected in the third nucleus **(right)**. Tightly associated Spelt1 and Spelt52 clusters are indicated with checkmarks. **(C)** Meiotic chromosomes at the anaphase I stage and interphase nuclei from the same cytological slide of the hybrid F2 genotype. Stretched Spelt1 fibers between homologous chromosomes are indicated with arrows. Scale bar = 10 μm.

In the maternal genotype from the population Katzir, there are 12 Spelt52 and 22 Spelt1 clusters on 14 individual chromosomes, or 14 Spelt1 and 7 Spelt52 clusters per seven bivalents, that is, when homologs are paired (Figure 3A, on the left). In somatic nuclei (Figure 3A, on the right), the number of both types of clusters vary and often is smaller than the number on 14 chromosomes (statistical analysis was not performed).

In the F1 hybrid genotype (Figure 3B; Raskina, 2017), maternal and paternal chromosomes are identified according to contrasting Spelt1 and Spelt52 patterning, as detailed in the small box. Maternal chromosomes carry 12 Spelt1 and 2 Spelt52 clusters; paternal chromosomes carry 3 Spelt1 and 5 Spelt52 clusters. In terms of the seven bivalents, there are 13 Spelt1 and 5 Spelt52 clusters in the genome. Somatic interphase nuclei from the anther tissues on the same cytological slide differ in the number of Spelt1 and Spelt52 clusters. Two nuclei (on the left and in the middle) are similar and contain 12 Spelt1 and 8 Spelt52 clusters, while in the third nucleus (on the right), the numbers of clusters are reduced due to associations.

In the F2 hybrid genotype (Figure 3C), the Spelt1 fibers between the homologous chromosomes at the stage of anaphase I are the consequences of meiotic recombination in the terminal chromosome regions; interchromosomal connections will be broken, and homologs will move to opposite pools with altered termini.

### Ectopic Associations of Somatic Chromosomes in Different Genotypes

**FIGURE 4:**
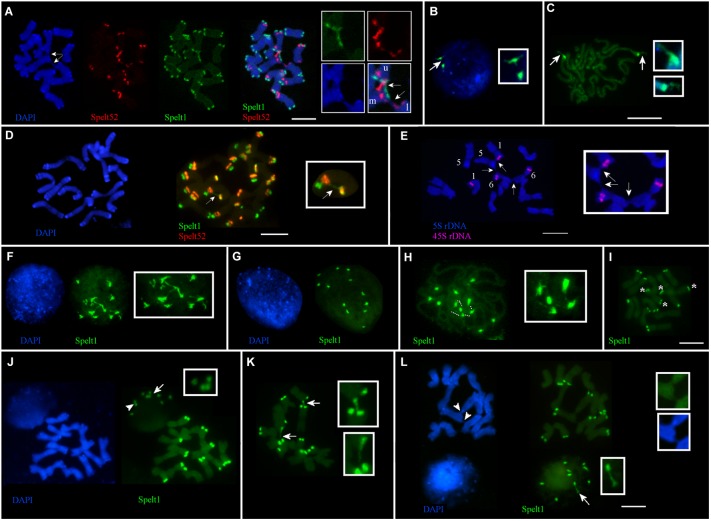
Cell-specific interchromosomal and intrachromosomal ectopic associations in different genotypes of *Ae. speltoides*. The chromosomes and nuclei from the seedling shoot apical meristems. **(A)** Genotype from Katzir population. Three chromosomes are involved in ectopic associations (arrows; enlargement in the small boxes). Specifically, Spelt1 and Spelt52 clusters of one of the two chromatids of the upper chromosome (this chromosome is indicated by the letter “u” in the small box) form ectopic fibers with similar clusters located on the one of two chromatids of the second chromosome (marked “m” in the small box), which is located in the middle. One chromatid of the third lower chromosome (marked “l” in the small box), which carries the Spelt52 cluster, forms ectopic fiber (visualized by DAPI staining) with the second chromatid of the “m” chromosome, and in this case, unknown sequences are involved in the ectopic association. **(B)** Interphase nucleus and **(C)** somatic prophase chromosomes of the Cankiri genotype. In the nucleus, one Spelt1 cluster is partially decondensed (arrow; enlargement in the small box). Both Spelt1 clusters on the prophase chromosomes are partially decondensed (arrows; enlargement in the small boxes). **(D)** Intrachromosomal ectopic association in the genotype from the Ramat haNadiv population. Ectopic Spelt1 fiber was revealed as a dotted line between the Spelt1 cluster of the short arm and Spelt52 cluster of the long chromosome arm (arrow; enlargement in the small box). This fiber consists of interspersed small green Spelt1 clusters and uncolored regions of unknown sequences. **(E)** Ectopic associations between somatic chromosomes bearing 5S rDNA and 45S rDNA clusters of plant from the Ankara population. One homolog of chromosome 5, one homolog of chromosome 1, and both homologs of chromosome 6 are involved in somatic associations (arrows; enlargement in the small box). Clusters of 5S rDNA and 45S rDNA appear intact; ectopic fibers are formed by some other sequences and revealed by DAPI staining. **(F–I)** Interphase nuclei and chromosomes from the same cytological slide of the hybrid genotype F2 (♀Cankiri × ♂Katzir). **(F)** Decondensed Spelt1 clusters interconnected by extended fibers. **(G)** Condensed clusters of Spelt1 form two groups of six clusters each in the nucleus. **(H)** Spelt1 clusters on prophase chromosomes are partially decondensed and interconnected by extended fibers (dashed lines; enlargement in the small box). **(I)** Metaphase plate: there are 12 Spelt1 clusters in the diploid genome. Two chromosomes carry Spelt1 clusters in both arms (asterisk), and 12 carry the cluster in the one arm. **(J–L)** Interphase nuclei and chromosomes from the same cytological slide of another hybrid genotype F2. **(J)** There are 10 Spelt1 clusters in diploid genome; none of the chromosomes contains clusters in both arms. In the interphase nucleus, associations of three (arrow; enlargement in the small box) and two clusters (arrowhead) are observed. **(K)** Ectopic associations between Spelt1 clusters of different chromosomes (arrows; enlargement in the small boxes) are revealed in a cell-specific manner. **(L)** Two Spelt1 clusters are connected by stretched fiber in the interphase nucleus (arrow, enlargement in the small box); ectopic associations between chromosomes are indicated with arrowheads (enlargement in two small boxes). Scale bar = 10 μm.

Frequent cell-specific interchromosomal somatic associations in apical shoot meristem were revealed in plants from different populations. Thus, in the Katzir genotype, among 32 metaphase plates analyzed on the same cytological slide, in 21 cases (65.6%), ectopic associations between chromosomes were detected in a cell-specific manner, regardless of the presence or absence of any tandem repeat clusters in the points of chromosomal connections (Figure 1D). Interchromatid somatic associations between three chromosomes were documented for the other genotype from the Katzir population (Figure 4A). In this instance, two chromatids of one chromosome are connected with single chromatids of two other chromosomes. Clusters of Spelt1 and Spelt52 are involved in one association and form interchromosomal fibers; in other association, the fiber between chromatids of two different chromosomes was revealed with DAPI staining.

No associations between the Spelt1 clusters were observed for homologous chromosomes 7 in the Cankiri plants. Partially decondensed Spelt1 clusters were documented in somatic nuclei (Figure 4B). Cell-specific alterations in Spelt1 clusters’ condensation/decondensation was observed in somatic prophase cells (Figure 4C).

In the genotype from the Ramat haNadiv population, intrachromosomal ectopic recombination was revealed (Figure 4D). Here, the Spelt1 fiber is observed as a dotted line, which may be an indication of intercalation/alternation of Spelt1 subclusters with some other type of uncolored DNA sequences or decondensation of uncolored regions. Ectopic associations between chromosomes 1 and 6, which carry 45S rDNA clusters, and chromosome 5, carrying 5S rDNA clusters, were documented for the genotype from the Ankara population (Figure 4E). Ectopic interchromatid fibers, which are revealed with DAPI staining, connect the short and/or long arms of somatic chromosomes, and the rDNA clusters appear intact.

In the F2 hybrid (♀Cankiri × ♂Katzir) genotype, at different interphase stages, 12 Spelt1 clusters were typically interconnected when decondensed (Figure 4F), and they apparently formed two groups of six clusters each when condensed, in the diploid nucleus (Figure 4G). On the prometaphase chromosomes (Figure 4H), partial decondensation and interconnections between five Spelt1 clusters were observed. In this genotype, two chromosomes carried Spelt1 clusters on both the short and long arms (Figure 4I). Therefore, the fibers between the four clusters in the prometaphase could be consequences of cell-specific ectopic associations between the short and long arms in the interphase, with the fifth cluster belonging to some other chromosome; alternatively, all five clusters could belong to different/nonhomologous chromosomes. However, in the other F2 hybrid genotype (Figures 4J–L) containing 10 Spelt1 clusters in the diploid genome, all chromosomes carry single Spelt1 clusters in one arm; while in the nuclei, associations of two and three clusters were revealed (Figures 4J,L). Two clusters may have belonged to homologous chromosomes, while three undoubtedly pointed to nonhomologous associations. In addition, ectopic chromatin fibers were detected between the chromosomal regions, and these did not carry tandem repeat clusters (Figure 4L). Altogether, the data obtained show that different chromosomes can be randomly involved in somatic associations.

Thus, tandem repeat cluster associations were revealed in nuclei at different interphase stages in both shoot apical meristems and anther’ somatic tissues. Ectopic associations between somatic chromosomes were documented in different original and hybrid genotypes. In microsporogenesis, nonhomologous/ectopic recombination was documented in different genotypes. As a possible result of broken associations, uncondensed/improperly packed DNA fibers, mainly in heterochromatic regions, were revealed in both the meiotic and somatic prophases-metaphases, especially in distal/terminal chromosomal regions enriched with different types of highly repetitive DNA.

## Discussion

The data obtained evidence on dynamic ectopic chromosomal interactions during somatic cell proliferation and differentiation and nonhomologous recombination in microsporogenesis of *Ae. speltoides*.

### Sequence- and Genotype-Specific Tandem Repeat Patterns and Dynamics Evidence on Homologous Chromosome Separation in Interphase Nuclei

The patterning of two types of tandem repeats, Spelt1 and Spelt52, changed dynamically in somatic interphase nuclei. In the nuclei at different interphase stages in various somatic tissues, the numbers of Spelt1 and Spelt52 clusters varied significantly, and they were mostly less than the numbers of clusters on the somatic and meiotic chromosomes. Associations of the clusters of the same type cause a reduction in their number; moreover, Spelt1 and Spelt52 tandem repeats formed joint clusters in nuclei. The reduction of the tandem repeat cluster number in the somatic nuclei may be the result of homologous and/or nonhomologous associations; in addition, intrachromosomal ectopic recombination could reduce the number of clusters. Individual genotypes from contrasting populations and intraspecific hybrids differed in the total abundance and chromosomal patterning of Spelt1 and Spelt52 and cluster dynamics in the interphase stage. In the Cankiri plants, distal regions of homologous chromosomes 7 generally separated in the nuclear space during the interphase. In parallel, in most cases, two 5S rDNA (located on chromosome 5) and four 45S rDNA (located on chromosomes 1 and 6) clusters also were separated in the nuclei. These data point to the separation of the homologous chromosomes of two subgenomes in the nucleus during the interphase stages, and they are consistent with data obtained in studies of other plant species, which have revealed largely random homologous and nonhomologous chromosome arrangements and a low frequency of pairing/associations in interphase nuclei (Pecinka et al., 2004; Schubert et al., 2007, 2014).

### The Dynamics of Spelt1 and Spelt52 Tandem Repeats Differ in the Interphase Nuclei

Associations of Spelt52 clusters were observed with a lower frequency than associations of Spelt1 clusters. The number of Spelt52 clusters in interphase nuclei mainly corresponded to the cluster number on the chromosomes. In the nuclei, both types of tandem repeat are usually separated due to decondensation of interspersed DNA sequences of other types. However, in this study, associated complex Spelt1–Spelt52 clusters were revealed in interphase nuclei in different genotypes. The highest frequency of cluster fusions was found in the Ramat haNadiv population, in which the abundances of both types of tandem repeats were highest in comparison with plants from the Cankiri and Katzir populations and hybrid genotypes (Raskina et al., 2011). The association of the Spelt1–Spelt52 clusters most likely indicated the elimination of intercluster sequences due to typically high frequencies of rearrangements in heterochromatic regions, specifically in the *Ae. speltoides* genome. However, we were unable to discriminate intrachromosomal associations and recombination events in nuclei, except when the number of colocalized clusters exceeded the number on the homologous chromosomes (Figures 4F,J).

### An Increase in the Number of Tandem Repeat Clusters in the Genome Causes an Increase in the Probability of Their Interactions

A comparison of contrasting genotypes indicated that, as the abundance of Spelt1 and Spelt52 tandem repeats increases in the genome, the frequency of cluster associations in the interphase also rises. In the Cankiri genotypes, a single Spelt1 cluster was found in approximately 3% of the nuclei; however, Spelt1 clusters associations was a common phenomenon in plants from Katzir and Ramat haNadiv. The associations of Spelt1 clusters exceeded the frequency of associations of Spelt52 clusters. At the same time, the two types of tandem repeats formed tightly associated complex clusters at different interphase stages.

Along with the dynamic change in the number of clusters as a result of their associations, partial decondensation of clusters in nuclei of different types was revealed. In the genome of *Ae. speltoides*, both types of tandem repeats are an integral part of heterochromatin, which is known to be condensed throughout the cell cycle, except for the time of replication in the late S-phase (Grewal and Moazed, 2003). The highest degree of associations of clusters of both types, with a decrease in their number and simultaneous decondensation have been observed in relatively small, dense and brightly DAPI-fluorescent nuclei (Figures 2C,E). Probably, these nuclei are at the stage of heterochromatin replication; however, this question remains open and requires additional study.

The decondensation of Spelt1 and Spelt52 clusters was also found in somatic nuclei of other types, which, according to their sizes and chromatin fiber compactness, were at the late interphase–early prophase stages (Figures 2A7,A8, G,H). Extended fibers between clusters of the same type and between Spelt1–Spelt52 clusters was most likely caused by ectopic interactions in the earlier interphase, and in turn, provide the proof for such events. At the metaphase stage, cell-specific ectopic associations between somatic chromosomes were revealed in all the investigated genotypes.

### Repetitive DNA Reshuffling Is Required for Genome Stabilization

In the interphase nuclei of hybrid genotypes, an increase in the number of Spelt1 clusters does not allow the discrimination of homologous chromosomes. Nevertheless, condensed Spelt1 clusters often form two spatially separated groups, which mirror the spatial arrangements of the terminal chromosomal regions of two subgenomes. In contrast, at the interphase stages, when the Spelt1 clusters are decondensed, they are usually interconnected by extended fibers, and the number of interconnected clusters evidences frequent nonhomological associations. It was shown for allopolyploid wheat that, in the somatic nuclei, homologous and nonhomologous chromosomes display non-random arrangement; different subgenomes occupy different territories, and homologous chromosomal sets are associated (Avivi and Feldman, 1980; Avivi et al., 1982).

Intraspecific hybrids, which were investigated in this work, were obtained by crossing genotypes with contrasting Spelt1 contents (Raskina, 2017). In the F1, homologous chromosomes preserved the parental patterns of tandem repeats. In contrast, the Spelt1 and Spelt52 clusters were almost evenly distributed between homologous chromosomes in F2 descendants (Figure 3). For cross-pollinated *Ae. speltoides*, heterozygosity for chromosomal rearrangements in distal/terminal regions is an inherent feature of individual plants in wild populations; however, homologous chromosomes demonstrate significant similarity in their heterochromatin patterns (Raskina et al., 2011). Presumably, an artificial asymmetry in tandem repeat patterns in the F1 intraspecific plant(s) could negatively affect the hybrid genomes’ stability, specifically, by altering the chromosome pairing and segregation. In addition, the subtelomeric location of Spelt1 may be tightly connected with telomere functioning and dynamics during both mitosis and meiosis (Bozza and Pawlowski, 2008; Tiang et al., 2012; Mainiero and Pawlowski, 2014). In the early meiotic prophase, homolog pairing is accompanied by repositioning of chromosomes in the nuclear space and “telomere bouquet” formation; and clustering of telomeres on the nuclear envelope may continue until early pachytene. Premeiotic and meiotic processes of telomere clustering involve subtelomeric DNA sequences (Bozza and Pawlowski, 2008), and the pattern and dynamics of Spelt1 in interphase nuclei may be associated with telomere dynamics and homologous recognition (Calderón et al., 2014; Sepsi et al., 2017).

Genome stabilization implies rearrangement/repatterning of heterochromatic clusters toward increasing similarity between homologs. Indeed, extended Spelt1 fibers between somatic and meiotic chromosomes is a common phenomenon for all the investigated genotypes. It can be assumed that the homogenization and stabilization of the genome, especially in the context of intraspecific hybrids, could be achieved gradually through mitotic cell proliferation and meiotic recombination, leading to heterochromatic cluster rearrangements in distal/terminal chromosomal regions. It may be proposed that cell-specific ectopic recombination events that occurred in premeiotic cell lineages result significant numbers of nonhomologous chromosomal associations detected in microgametogenesis in *Ae. speltoides*.

### Nonhomologous Meiotic Chromosome Associations May Be Consequences of Erroneous DNA Replication and Repair in Premeiotic Somatic Cell Lineages and/or Ectopic Meiotic Recombination

The spatial distributions of individual chromosome territories in interphase nuclei is tightly related with processes of DNA transcription, repair and recombination (Fransz and De Jong, 2011; Schubert and Shaw, 2011; Hübner et al., 2013). Chromosomal rearrangements and alterations in chromosome structure are the direct consequences of errors occurring in the processes of DNA replication and repair (Andersen and Sekelsky, 2010; Lambert et al., 2010; Borde and de Massy, 2013). Specifically, in the cases of DNA double-strand breaks (DSBs) when homologous DNA strands are unavailable, the same or similar ectopic sequences may serve as the source for DNA repair (Puchta, 2005; Wicker et al., 2010; Anand et al., 2014; Knoll et al., 2014). Homologous and nonhomologous/ ectopic associations may occur between mitotic chromosomes (Godin and Stack, 1976; Klasterska, 1978; Lavania and Sharma, 1978; Pedrosa et al., 2001; Ghosh, 2003) in interphase plant nuclei between spatially distant regions (Schubert et al., 2014). As homologs are separated in the nucleus, nonhomologous chromosomes appear in closer proximity to each other in the subgenome, and ectopic sequences serve as templates in the replication/reparation processes.

In large genomes of cereals, chromosomes display a Rabl orientation in the interphase nuclei, when telomeres and centromeres cluster in opposite nucleus pools. It is assumed that this arrangement is a consequence of the preceding anaphase (Scherthan, 2001). The karyotype of *Ae. speltoides* is composed of metacentric/slightly submetacentric chromosomes that are similar in size, and the terminal regions of short and long chromosome arms appear in close proximity in the anaphase stage. Seemingly, in *Ae. speltoides*, in each of the subgenomes and between the subgenomes, ectopic recombination may often occur randomly, as was shown for *Arabidopsis* (Schubert et al., 2007, 2012, 2014). This is evidenced by the low frequency of homologous chromosome associations in Cankiri plants. In addition, intrachromosomal recombination may be the reason for the tandem repeat cluster association/intercalation, along with the interchromosomal ectopic associations in mitosis.

Ectopic chromatin fibrils were revealed not only between Spelt1 and Spelt52 clusters, but also between distal/terminal and intercalary chromosomal regions. Plant genomes are enriched with various types of repetitive DNA, and TEs are the prevailing genomic fraction in *Ae. speltoides* (Middleton et al., 2013). The ubiquitous distribution of TEs determines the probability of ectopic recombination between almost any parts of the plant chromosome, and nonhomologous meiotic recombination has been shown for *Ae. speltoides* previously (Raskina, 2017). Often, illegitimate recombination is observed in the heterochromatin regions, comprising various repetitive DNA types, primarily tandem repeats and TEs.

During the interphase stage, the spatial distribution of chromosomes in the nucleus changes (Avivi and Feldman, 1980; Schubert et al., 2014). The movements in the nucleus and simultaneous condensation of the chromosomes toward the metaphase stage appear to be accompanied by breaks of numerous ectopic associations. In the early meiotic prophase in different species, heterochromatic clusters are dynamically associated and dissociated (Bozza and Pawlowski, 2008), and chromosome movements may be the mechanisms involved in the rupturing of interchromosomal associations and entanglements (Koszul and Kleckner, 2009). However, numerous intra- and interchromosomal fibers are detected at the stages of somatic metaphase and until late meiosis in *Ae. speltoides*. When these links are broken, at all stages of cell proliferation and differentiation, significant portions of chromatin—especially in distal/terminal heterochromatic chromosomal regions—appear to be damaged and improperly packed/decondensed. This could provoke chromosomal aberrations in the following cell cycle. However, chromosome rearrangements contribute to heterochromatin cluster repatterning and genome stabilization, as was discussed above for intraspecific hybrids. In the ontogenesis of contrasting genotypes, the overall abundance/copy numbers and structural integrity of certain DNA sequences changed tissue-specifically (Shams and Raskina, 2018), and significant differences between individual plants of *Ae. speltoides* were documented.

Altogether, the obtained data showed that perpetual intraorganismal reshuffling of repetitive DNA mirrors the structural plasticity of the *Ae. speltoides* genome, which is interlinked with genetic diversity through the species distribution area in contrasting ecogeographical environments in and around the Fertile Crescent.

## Author Contributions

EZ, YP, and OR conducted confocal and electron microscope imaging and analyzed the data. OR conducted the experiments and drafted the manuscript. All of the authors read and approved the manuscript.

## Conflict of Interest Statement

The authors declare that the research was conducted in the absence of any commercial or financial relationships that could be construed as a potential conflict of interest.

## Footnotes

1 https://imagej.net/Fiji
