# Supplementary material for: Repetitive DNA in the Architecture, Repatterning, and Diversification of the Genome of Aegilops speltoides Tausch (Poaceae, Triticeae)
Source: Front Plant Sci. 2018 Dec 4;9:1779. doi: 10.3389/fpls.2018.01779 (PMC6288716; doi:10.3389/fpls.2018.01779)
Supplement: Supplementary file 1 [file Presentation_1.pptx]

## Slide 1
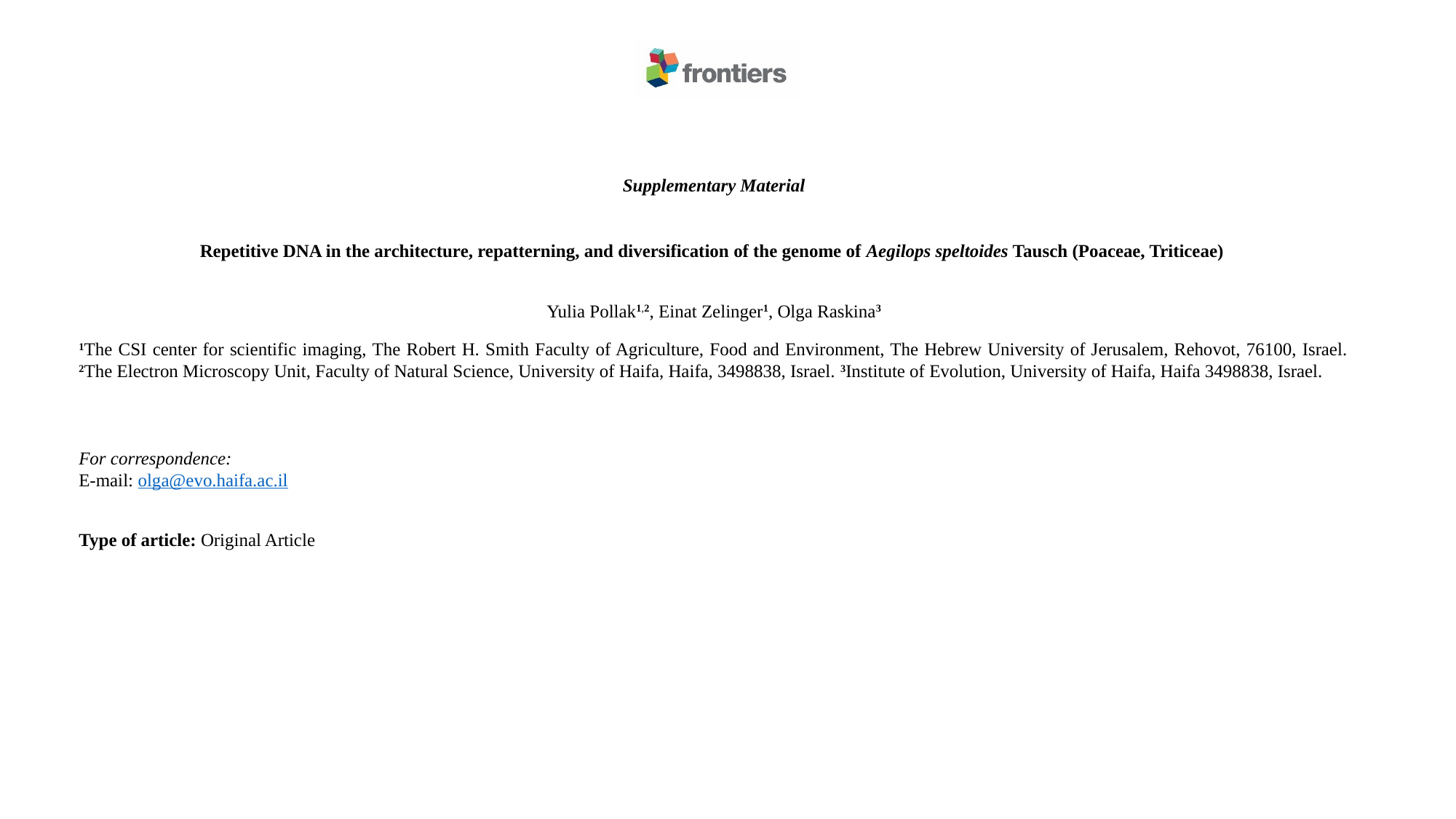

Supplementary Material
Repetitive DNA in the architecture, repatterning, and diversification of the genome of Aegilops speltoides Tausch (Poaceae, Triticeae)
Yulia Pollak1,2, Einat Zelinger1, Olga Raskina3
1The CSI center for scientific imaging, The Robert H. Smith Faculty of Agriculture, Food and Environment, The Hebrew University of Jerusalem, Rehovot, 76100, Israel. 2The Electron Microscopy Unit, Faculty of Natural Science, University of Haifa, Haifa, 3498838, Israel. 3Institute of Evolution, University of Haifa, Haifa 3498838, Israel.
For correspondence:
E-mail: olga@evo.haifa.ac.il
Type of article: Original Article

## Slide 2
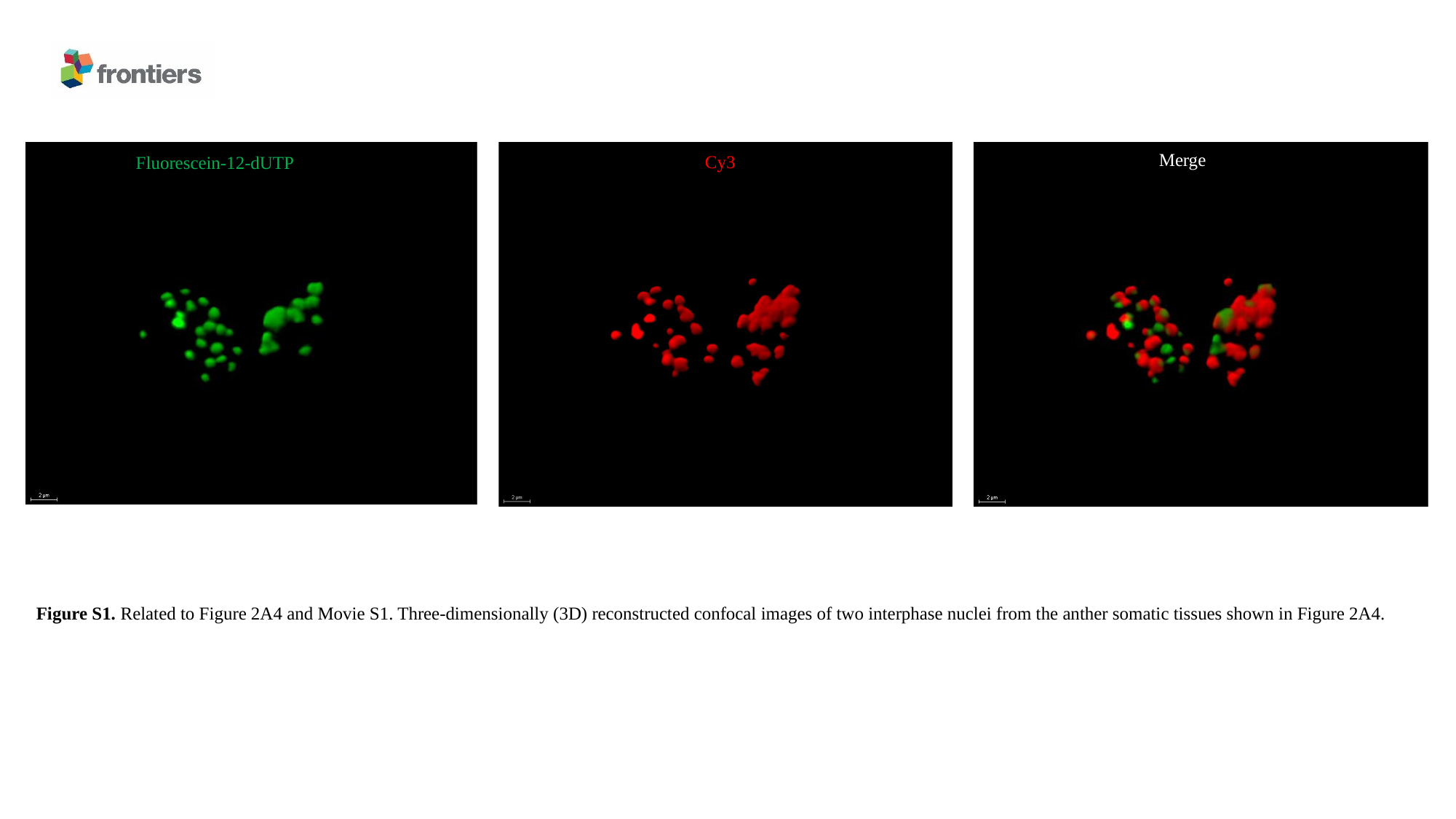

Merge
Cy3
Fluorescein-12-dUTP
Figure S1. Related to Figure 2A4 and Movie S1. Three-dimensionally (3D) reconstructed confocal images of two interphase nuclei from the anther somatic tissues shown in Figure 2A4.

## Slide 3
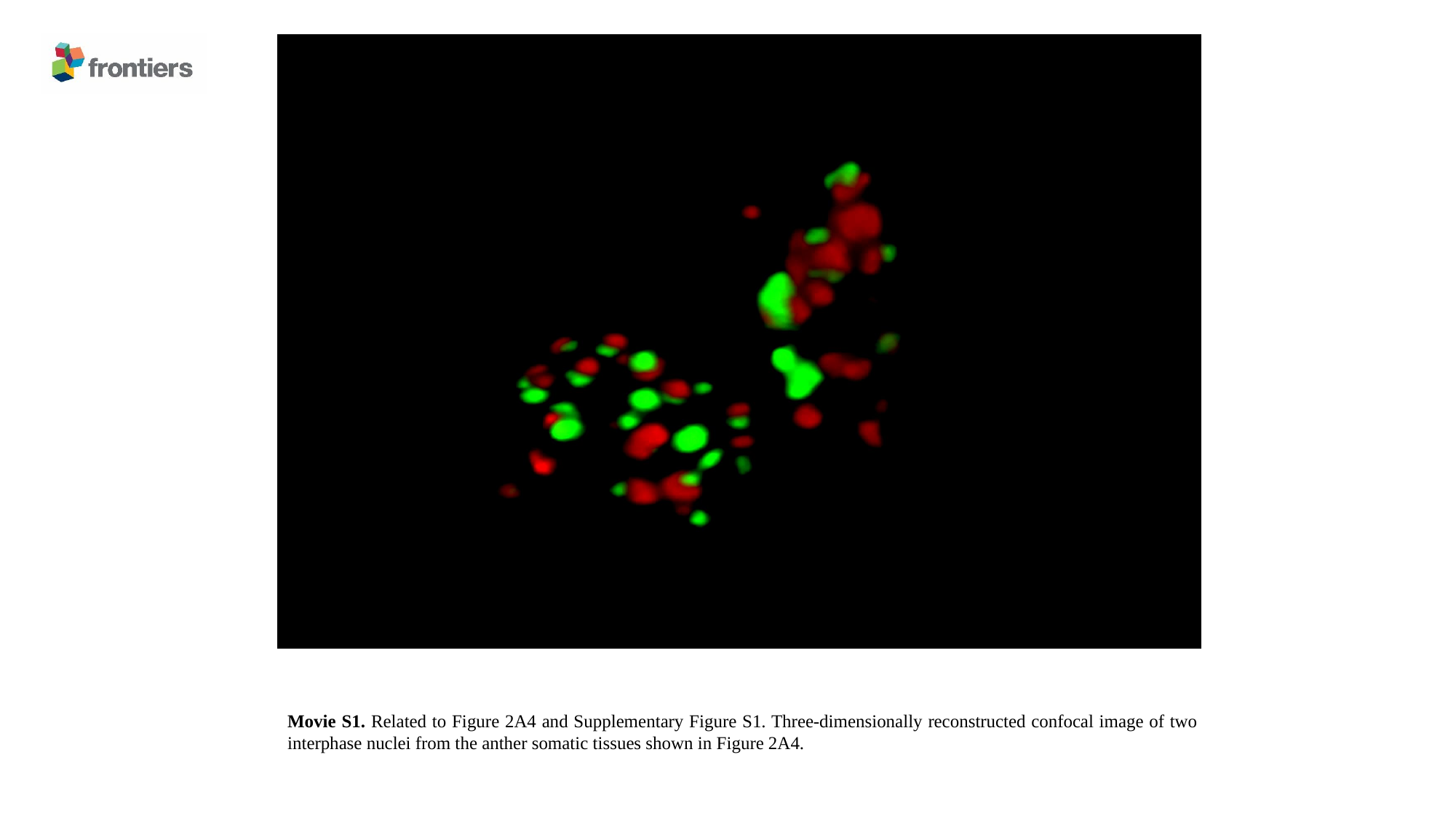

Movie S1. Related to Figure 2A4 and Supplementary Figure S1. Three-dimensionally reconstructed confocal image of two interphase nuclei from the anther somatic tissues shown in Figure 2A4.

## Slide 4
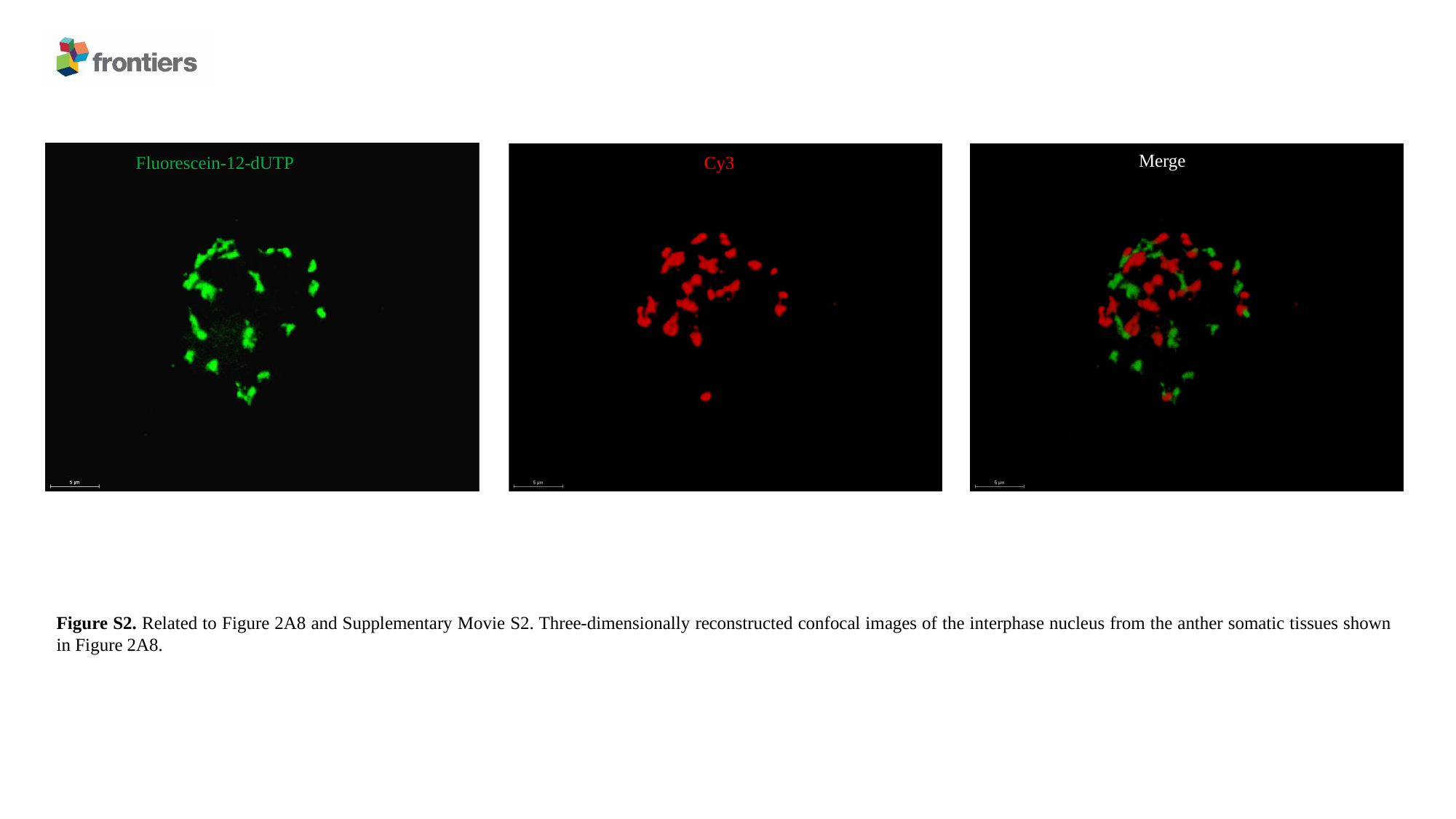

Merge
Cy3
Fluorescein-12-dUTP
Figure S2. Related to Figure 2A8 and Supplementary Movie S2. Three-dimensionally reconstructed confocal images of the interphase nucleus from the anther somatic tissues shown in Figure 2A8.

## Slide 5
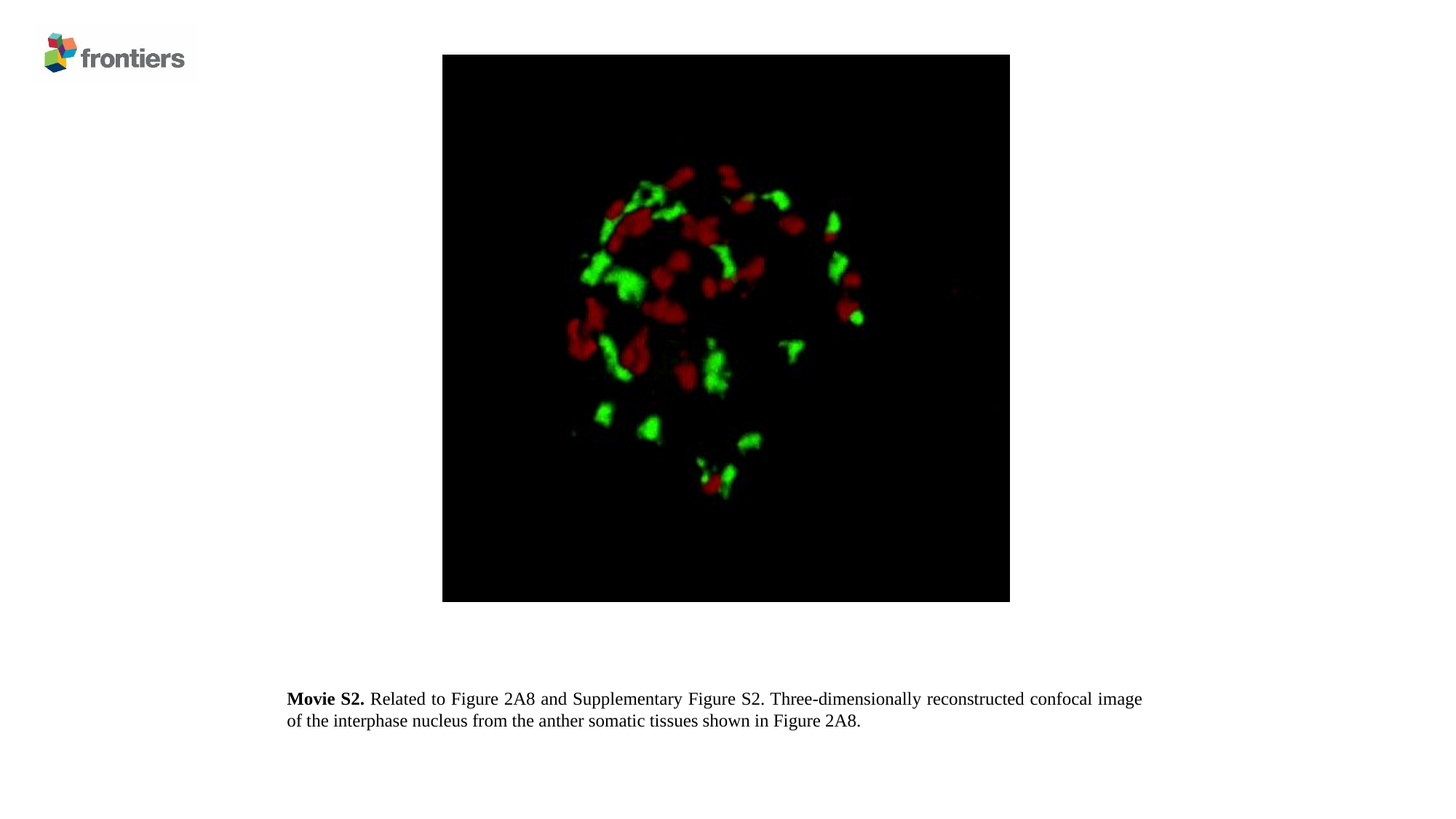

Movie S2. Related to Figure 2A8 and Supplementary Figure S2. Three-dimensionally reconstructed confocal image of the interphase nucleus from the anther somatic tissues shown in Figure 2A8.

## Slide 6
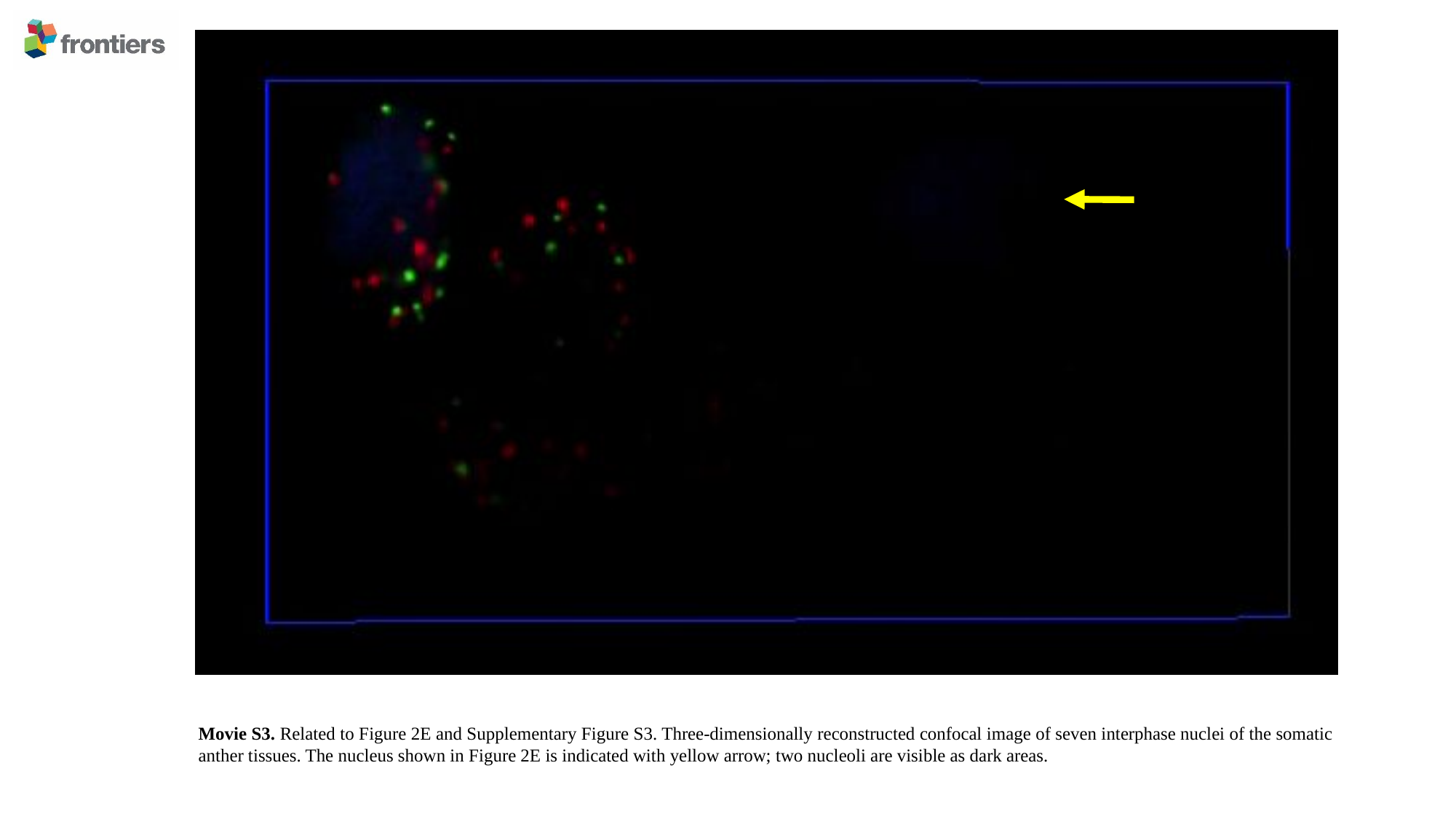

Movie S3. Related to Figure 2E and Supplementary Figure S3. Three-dimensionally reconstructed confocal image of seven interphase nuclei of the somatic anther tissues. The nucleus shown in Figure 2E is indicated with yellow arrow; two nucleoli are visible as dark areas.

## Slide 7
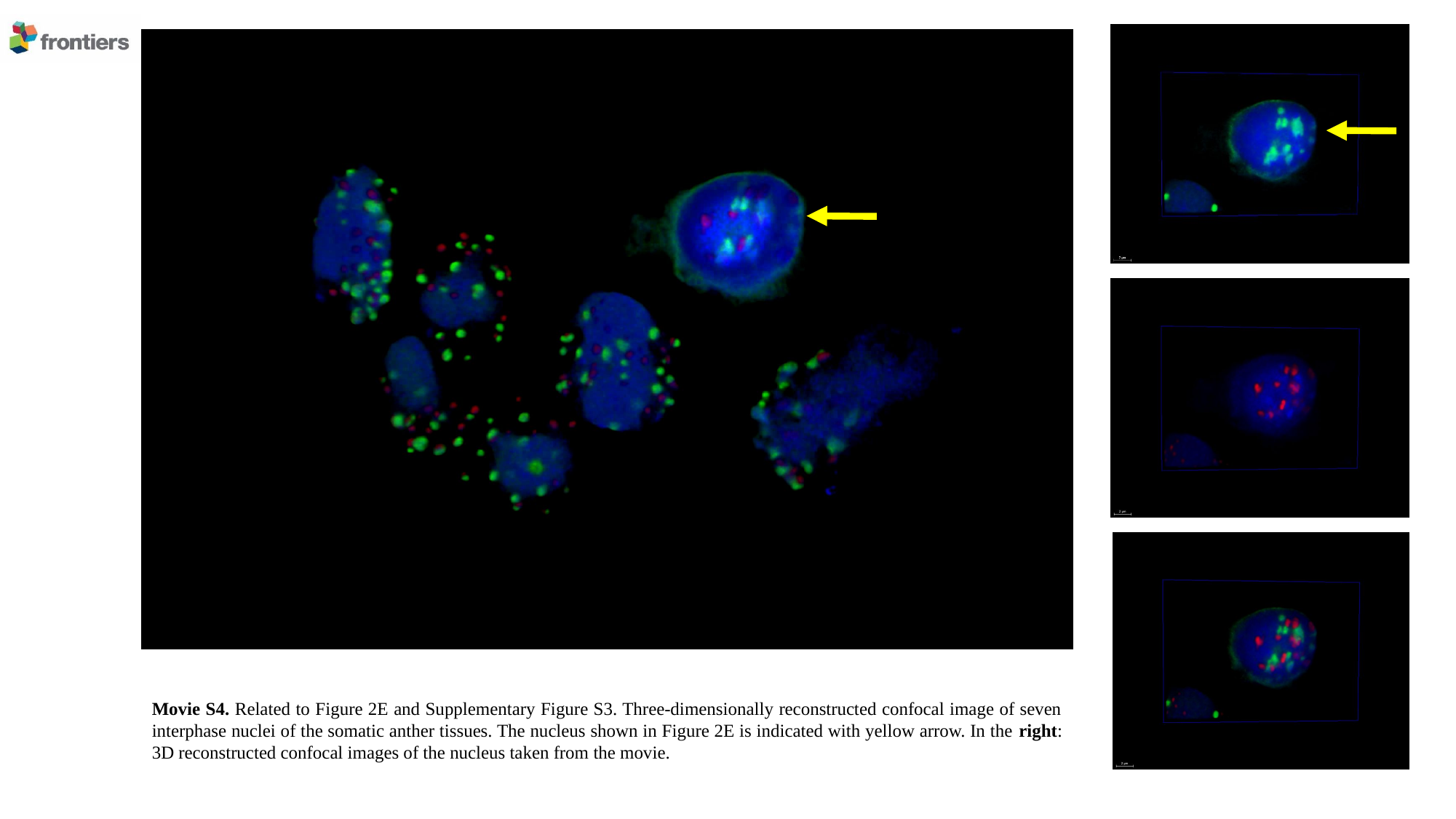

Movie S4. Related to Figure 2E and Supplementary Figure S3. Three-dimensionally reconstructed confocal image of seven interphase nuclei of the somatic anther tissues. The nucleus shown in Figure 2E is indicated with yellow arrow. In the right: 3D reconstructed confocal images of the nucleus taken from the movie.

## Slide 8
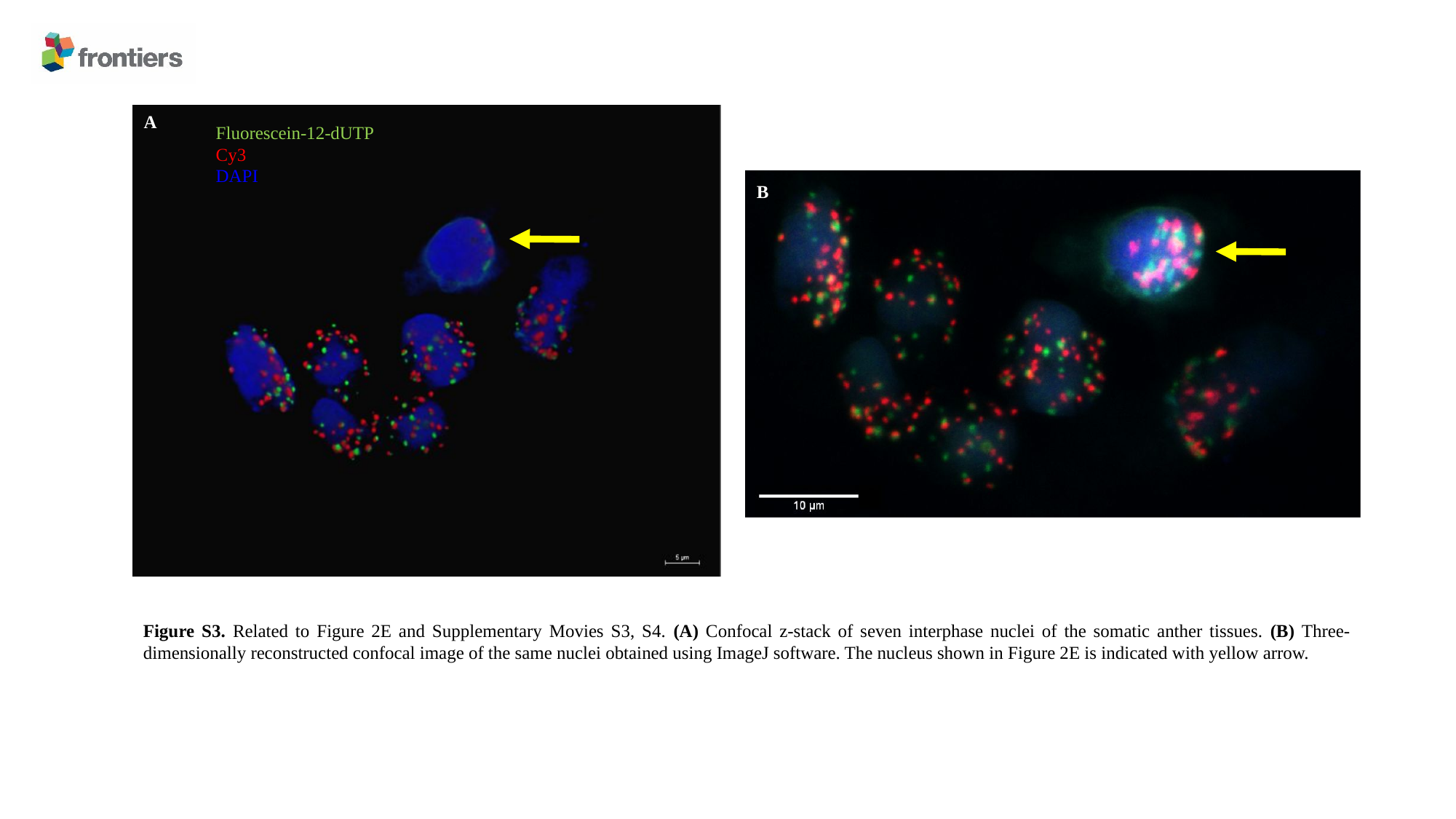

A
Fluorescein-12-dUTP
Cy3
DAPI
B
Figure S3. Related to Figure 2E and Supplementary Movies S3, S4. (A) Сonfocal z-stack of seven interphase nuclei of the somatic anther tissues. (B) Three-dimensionally reconstructed confocal image of the same nuclei obtained using ImageJ software. The nucleus shown in Figure 2E is indicated with yellow arrow.

## Slide 9
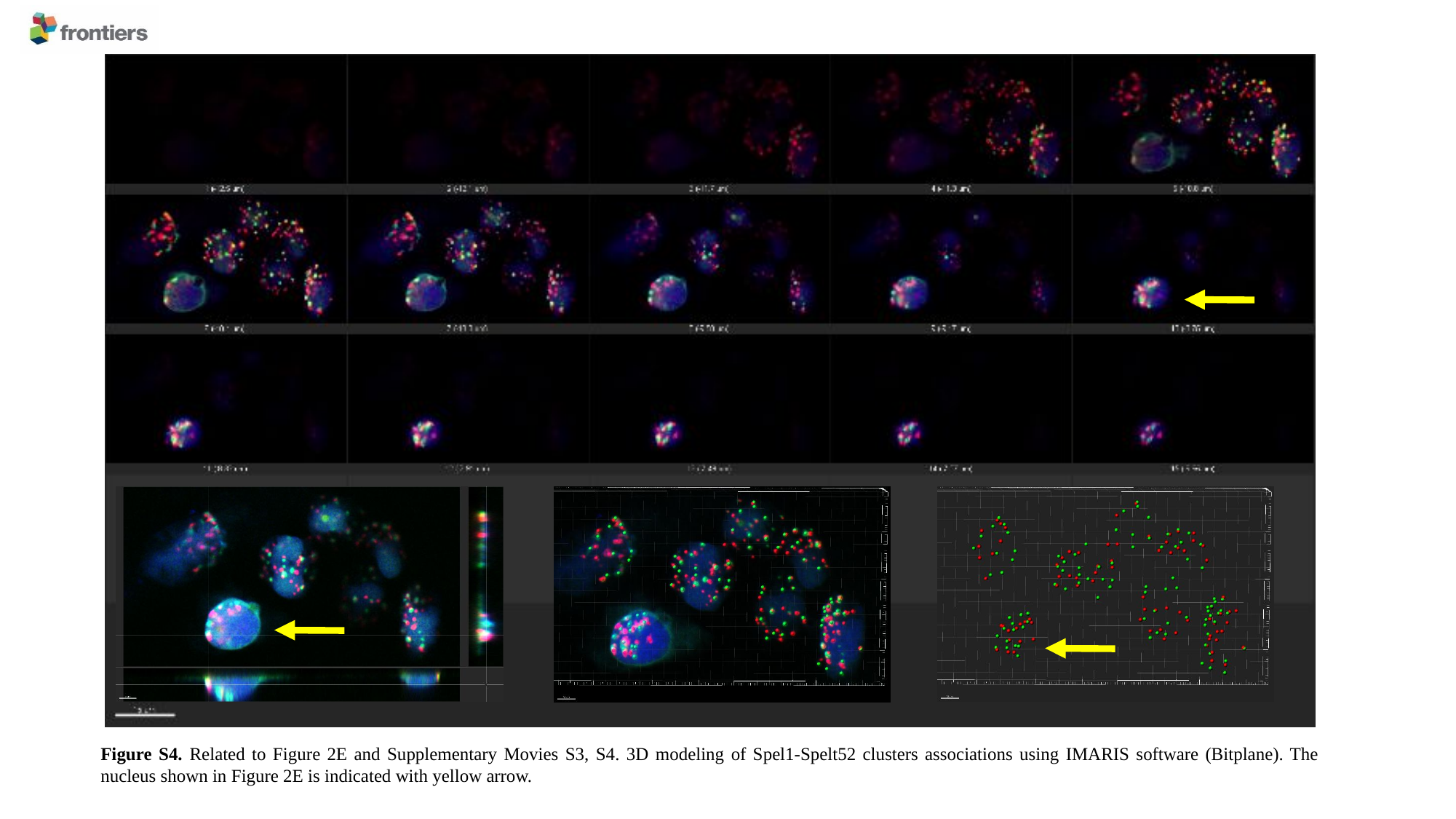

Figure S4. Related to Figure 2E and Supplementary Movies S3, S4. 3D modeling of Spel1-Spelt52 clusters associations using IMARIS software (Bitplane). The nucleus shown in Figure 2E is indicated with yellow arrow.
